# Mycoplasma hominis Intracranial Abscess Diagnosed by Characteristic Colonies Obtained Through Extended Culture: Case Report and Literature Review

**DOI:** 10.7759/cureus.79981

**Published:** 2025-03-03

**Authors:** Oki Sato, Keitaro Iwata, Makoto Murase, Eiyu Ebata, Kiyofumi Ohkusu, Masakazu Sasaki, Daisuke Ono

**Affiliations:** 1 Infectious Diseases, Saitama Medical Center/Saitama Medical University, Saitama, JPN; 2 Emergency Medicine, Saitama Medical Center/Saitama Medical University, Saitama, JPN; 3 Microbiology, Saitama Medical Center/Saitama Medical University, Saitama, JPN; 4 Microbiology, Tokyo Medical University, Tokyo, JPN; 5 Laboratory Medicine, Toho University Omori Medical Center, Tokyo, JPN

**Keywords:** 16s rrna gene, characteristic colony, extended culture, intracranial abscess, mycoplasma hominis

## Abstract

*Mycoplasma hominis (M. hominis)* causes genitourinary infections and pregnancy-related complications. Reports of intracranial abscesses due to *M. hominis* infection are rare. Here, we report a *M. hominis* intracranial abscess case following a traffic accident who was admitted to our hospital (day 0). The patient, a man in his 70s, underwent cystourethrography, and a urethral catheter was inserted. On day three, the patient underwent intracerebral hematoma evacuation, and on day seven, intravenous ceftazidime and vancomycin were administered after the patient developed a fever. On day 10, the antibiotics were switched to meropenem and vancomycin due to persistent fever. On day 17, magnetic resonance imaging revealed brain and epidural abscesses, and abscess drainage was performed. Gram staining of the abscess specimen showed numerous polymorphonuclear leukocytes, but no visible microorganisms. On day 19, two days after inoculating the culture, tiny pinpoint colonies were observed on the blood agar. Sequencing of the 16S rRNA gene from these colonies revealed the presence of *M. hominis*. On day 27, the treatment was changed to levofloxacin and clindamycin for treatment of the intracranial abscesses caused by *M. hominis*. Antibiotic therapy was continued for an additional 52 days until the abscesses disappeared. No recurrences were observed. When bacteria are suspected to be the cause of an intracranial abscess with a risk of *M. hominis* infection, and Gram staining does not show any microorganisms, considering *M. hominis* as one of the causative pathogens, conducting extended culture is important.

## Introduction

*Mycoplasma hominis* is a commensal bacterium of the genitourinary and respiratory tracts [1]. It has been associated with genitourinary infections, pregnancy-related complications, and occasionally postoperative wound infections following open-heart surgeries, as well as septic arthritis and pneumonia.　

Reports of intracranial abscesses due to *M. hominis* infection are rare, with only 19 cases reported to date (one of the case reports presented two different cases) [2-19]. Many cases were diagnosed based on the characteristic colony observed through extended culture. Here, we report a case of intracranial abscess diagnosed in a patient following a traffic accident based on the characteristic colony morphology observed through extended culture.

## Case presentation

**Figure 1:**
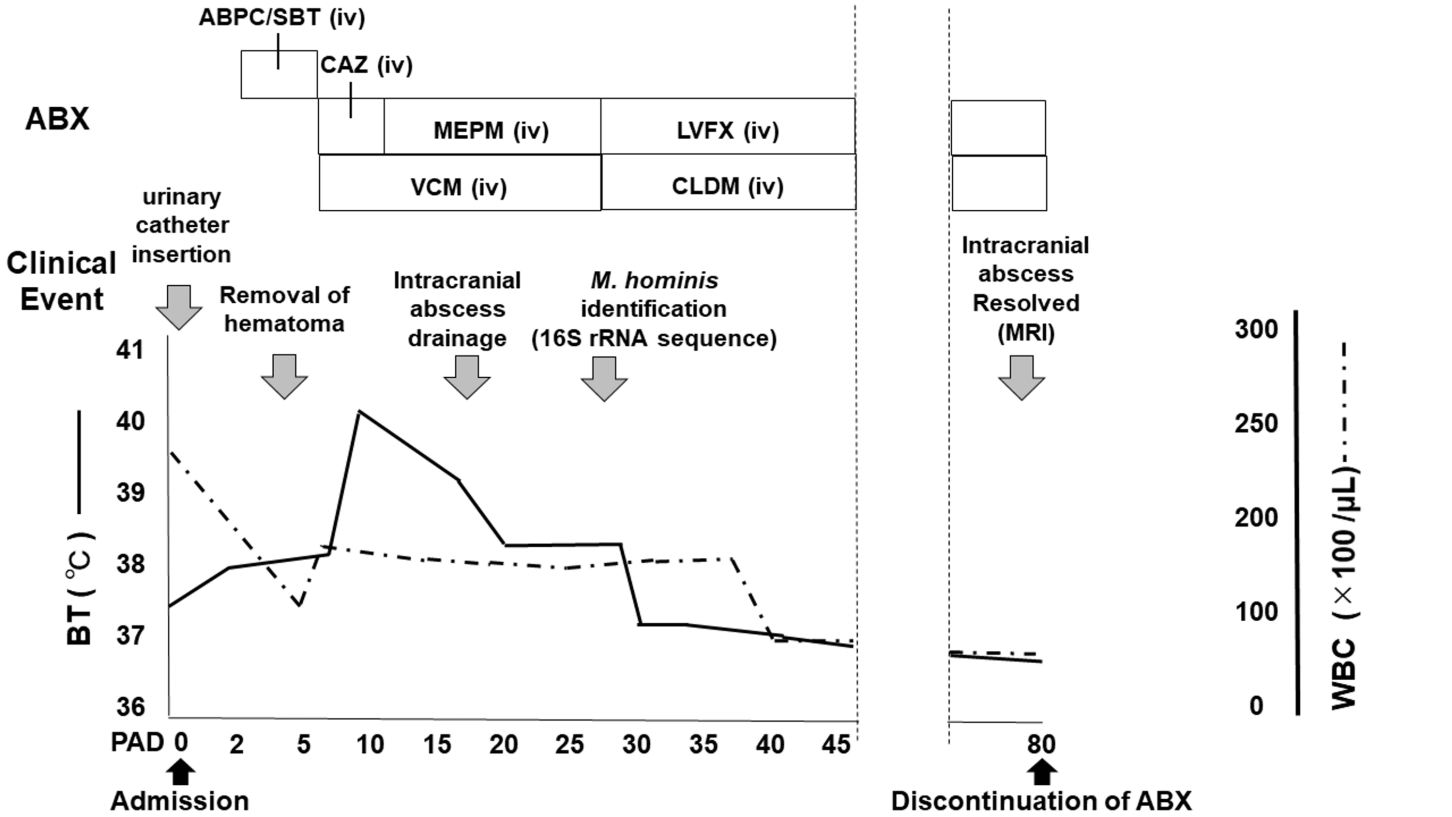
Clinical course of the patient. ABX: antibiotics; ABPC/SBT: ampicillin/sulbactam; CAZ: ceftazidime; MEPM: meropenem; VCM: vancomycin; LVFX: levofloxacin; CLDM: clindamycin; iv: intravenous; 16S rRNA: 16s ribosomal ribonucleic acid; MRI: magnetic resonance imaging; BT: blood temperature; WBC: white blood cell; PAD: post-admission days.

A previously healthy man in his 70s was admitted to our hospital following a traffic accident (day 0) (Figure 1). The patient showed a reduced level of alertness [Glasgow coma scale (GCS): E4V4M5] and required intubation because he was disturbed, complicating medical treatments. Computed tomography revealed a brain contusion, subdural hematoma, facial fractures, and pubic and sciatic fractures. In addition, the patient underwent cystourethrography for gross hematuria and hematoma around the bladder, and a urethral catheter was inserted. On day three, the intracerebral hematoma was drained. On the same day, intravenous ampicillin/sulbactam (ratio 2:1, 3 g) was administrated every six hours for suspected pneumonia.

By day seven, his body temperature had increased. The vital signs were as follows: consciousness, GCS E3VTM5; body temperature, 39.6 °C; blood pressure, 137/70 mmHg; pulse rate, 102 beats/min; respiratory rate, 24 beats/min; and oxygen saturation, 97 % (intubated, FiO2 30 %). Physical examination revealed swelling of the left face. Blood tests showed an elevated white blood cell (WBC) count (13,700/uL)　and C-reactive protein (25.5 mg/dL) (Table 1). A lumbar puncture was performed, and the cerebrospinal fluid (CSF) analysis showed red blood cell 16,909/mm³, WBC 385/mm³, and glucose 48 mg/dL (plasma glucose 111 mg/dL). The Gram staining of the CSF after centrifugation, showed no microorganisms. Later, blood and CSF cultures were found to be negative. As meningitis was suspected, treatment with intravenous ceftazidime (2 g every eight hours) and vancomycin (1.5 g every 12 h) was initiated (day seven) (Figure 1). On day 10, the antibiotics were switched to intravenous meropenem (2 g every eight hours) plus vancomycin because the fever had not subsided and the laboratory data showed no improvement.

**Table 1:** Laboratory findings (day 7).

Parameters	Patient Values	Reference Range
Blood Test		
White blood cells (×1000/μL)	13.7	3.3 – 8.6
Red blood cells (×10,000/μL)	308	435 - 555
Hemoglobin (g/dL)	9.4	13.7 – 16.8
Hematocrit (%)	29.7	40.7 - 50.1
Platelets (×10,000/μL)	243	150 - 400
Sodium (mmol/L)	138	138 - 145
Potassium (mmol/L)	4.1	3.6 – 4.8
Chloride (mmol/L)	106	101 - 108
Albumin (g/dL)	2.6	4.1 - 5.1
Blood urea nitrogen (mg/dL)	19	8 - 20
Creatinine clearance (mg/dL)	0.74	0.65 - 1.07
Aspartate transaminase (units/L)	44	13 - 30
Alanine transaminase (units/L)	46	10 - 42
Alkaline phosphatase (units/L)	107	38 - 113
C-reactive protein (mg/dL)	25.5	< 0.014
Plasma glucose (mg/dL)	111	70 - 109
Cerebrospinal Fluid Test		
Red blood cells (/mm³)	16,909	0
White blood cells (/mm³)	385	< 5
Glucose (mg/dL)	48	50 - 80

**Figure 2:**
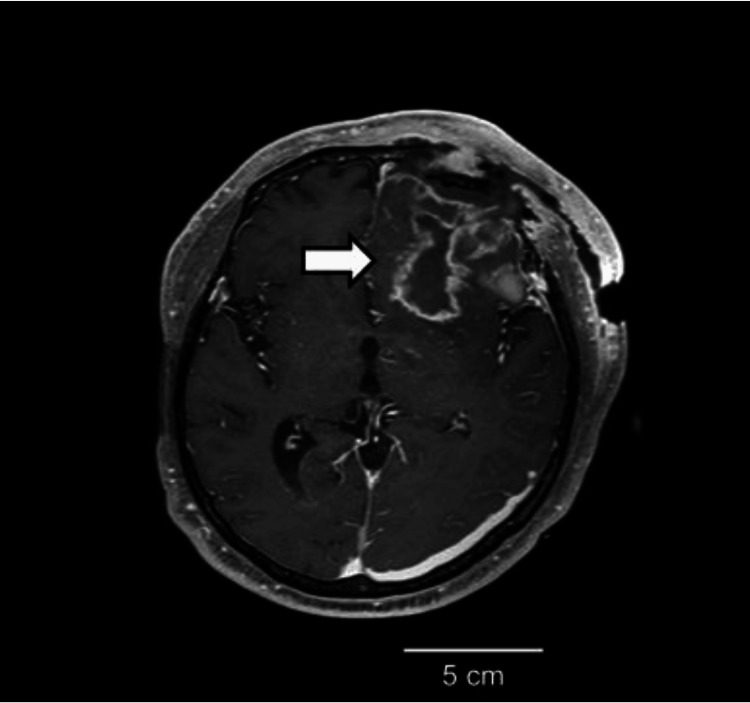
Magnetic resonance imaging (MRI) findings (day 17). The MRI on day 17 revealed a lesion (arrow) with high signal intensity around the hematoma removal site, as observed on contrast-enhanced T1-weighted images.

On day 17, magnetic resonance imaging (MRI) of the head was performed, and contrast-enhanced T1-weighted images detected a lesion with high signal intensity around the hematoma removal site (Figure 2), suggesting brain and epidural abscesses. After examining the images, abscess drainage was performed. However, Gram staining of the abscess specimen showed numerous polymorphonuclear leukocytes, but no microorganisms were detected.

**Figure 3:**
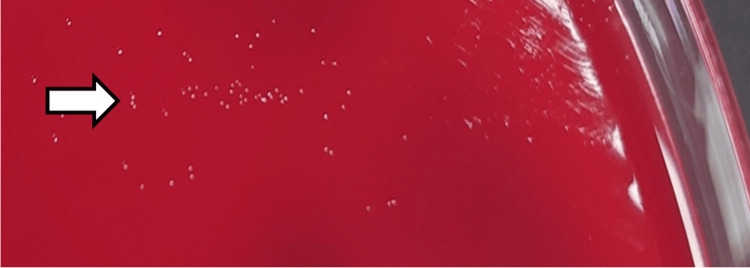
Tiny pinpoint colonies of Mycoplasma hominis (arrow) after a 2-day culture on blood agar.

On day 19, two days after incubation, tiny pinpoint colonies were observed on the blood agar (Figure 3). The colonies were used to perform tests such as matrix-assisted laser desorption/ionization time-of-flight mass spectrometry (MALDI-TOF MS), but the strain could not be identified. Based on the characteristic appearance of the colonies on blood agar, *Mycoplasma hominis* was suspected, and 16S rRNA gene analysis was performed. Sequencing of the 16S rRNA gene from the colonies revealed 100 % (1440/1440) homology with *M. hominis *PG21^T^ (accession number: FP236530). After the results of the 16S rRNA gene sequencing, the antibiotics were switched to levofloxacin (500 mg every 24 h) and clindamycin (900 mg every eight hours) on day 27, based on previous reports of *M. hominis* intracranial abscesses. Owing to poor colony growth, a minimum inhibitory concentration (MIC) test could not be performed.

On day 29, the patient became afebrile. Blood tests revealed a decreased WBC count to the normal range. Antibiotic administration was continued for an additional 52 days until the abscess disappeared, as confirmed by a follow-up MRI. No recurrence was observed at the time of writing (at least one year).

## Discussion

Here, we report a case of *M. hominis* intracranial abscess diagnosed using its characteristic “fried egg” colony morphology obtained through extended culture. *M. hominis* is a normal flora of the human urogenital tract and is found more frequently in sexually active individuals [20]. As far as our literature search revealed, only 19 cases of *M. hominis* intracranial abscess have been previously reported [2-19]. Several points need to be considered.

First, several risk factors for *M. hominis* intracranial abscess exist (Table 2). These risk factors include direct invasion from trauma or surgery, bacteremia due to urological or gynecological procedures such as childbirth or urinary catheter placement, and the presence of hematomas or hemangiomas [7, 12, 17]. In this case, risk factors, such as trauma, surgery, urinary catheter placement, and hematoma were identified. Blood cultures were negative in this case, and *M. hominis* growth can be inhibited by sodium polyanetholesulfonate, which is commonly supplemented in blood culture bottles [21]. This may account for the false-negative results obtained in the blood cultures in this case.

**Table 2:** Summary of the clinical and microbiologic features of Mycoplasma hominis intracranial abscess cases from literature. * One of the case reports Ref. No [10] presented two different cases.

Literature [Ref. No in this report]	Reported year	Sex	Age	Reported risks for M. hominis brain abscess	M. hominis identification triggered from the characteristic colony	Days to the colony formation (intracranial abscess specimen)	Definitive identifications	Antibiotics used for M. hominis brain abscess
Siber et al. [2]	1977	male	0	neonate	yes	no description （detected in CSF in 3 days）	antibody test	chloramphenicol
Payan et al. [3]	1981	male	29	trauma, surgery, hematoma	yes	2	agar technique and growth inhibition test	tetracycline
Kersten et al. [4]	1995	male	20	trauma, urinary catheter, hematoma	yes	5	agar technique	doxycycline and clindamycin
Zheng et al. [5]	1997	female	22	childbirth, surgery, hematoma	yes	4	immunofluorescence test and immunoblot assay	no description
Rao et al. [6]	2002	male	0	neonate	yes	4	16S rRNA	doxycycline and clindamycin
House et al. [7]	2003	female	40	vulvar irritation and ulceration, hemangiomas	yes	4	16S rRNA	ciprofloxacin
Douglas et al. [8]	2003	female	17	trauma, surgery, childbirth, hematoma	yes (isolated from CSF）	no description （detected in CSF in ten days）	polymerase chain reaction and nucleic sequencing	doxycycline and clindamycin
Kupila et al. [9]	2006	male	40	trauma, cystoscopy, and urinary catheter	no	no description	polymerase chain reaction and nucleic sequencing	tetracycline
McCarthy et al. [10] *	2008	male	48	surgery	no	no description	16S rRNA	gatifloxacin and clindamycin
McCarthy et al. [10] *	2008	female	17	trauma, surgery	no	no description	no description	gatifloxacin
Chong et al. [11]	2009	female	0	neonate, hematoma	no	no description	IST2kit	minocycline
Al Masalma et al. [12]	2011	female	41	uterus curettage, surgery, hematoma	no	no description	16S rRNA	doxycycline
Henao-Martínez et al. [13]	2012	male	40	trauma, surgery, hematoma	yes	no description	16S rRNA	doxycycline
Pailhoriès et al. [14]	2013	male	43	trauma, surgery, urinary catheter, hematoma	yes	4	TOF-MS and 16S rRNA	doxycycline and levofloxacin
Bergin et al. [15]	2017	male	57	surgery, urinary catheter, hematoma	no	5	16S rRNA	doxycycline
Qamar et al. [16]	2021	female	25	trauma, surgery, hematoma	yes	4	16S rRNA	doxycycline and levofloxacin
Potruch et al. [17]	2022	male	25	trauma, surgery, hematoma	yes (isolated from CSF)	no description （detected in CSF in two days）	TOF-MS and 16S rRNA	doxycycline
Chen et al. [18]	2023	male	52	trauma, hematoma	yes	no description	16S rRNA	minocycline
Lee et al. [19]	2024	male	52	surgery, urinary catheter	no	no description	16S rRNA	levofloxacin
This case	2024	male	71	trauma, surgery, urinary catheter, hematoma	yes	2	16S rRNA	levofloxacin and clindamycin

Second, it is a challenge to identify Mycoplasma species by Gram staining, as they are not visible using this staining technique. Proper identification of Mycoplasma species on culture media requires special growth media, such as pleuropneumonia-like organism (PPLO) agar [22]. Although colonies of *M. hominis* can grow on blood agar medium, a selective medium such as PPLO agar is required for enhanced growth. In Japan, PPLO medium is not commonly used in standard laboratories. In addition, the colonies of *M. hominis* are small and dotted with a dense center, often described as a “fried egg”. Of the 19 case reports of *M. hominis* intracranial abscess [2-19], these characteristic colonies were identified in approximately 60 % of the identifications of *M. hominis* intracranial abscess cases (Table 2). Thus, careful observation of colonies is important for identifying *M. hominis*. In this case, *M. hominis* was suspected because of the characteristic “fried egg” colonies and the absence of visible bacteria by Gram staining owing to the cell wall deficit in *M. hominis*. TOF-MS could not identify the strain. In addition, because our microbiology laboratory does not routinely use PPLO medium and the colonies failed to grow further on the blood agar, a minimum inhibitory concentration (MIC) test could not be performed. For the conclusive identification of *M. hominis*, 16S rRNA gene sequencing is frequently used, as in this case. In the previous 19 cases (one of the case reports presented two different cases), identifications of nine cases were conducted based on 16S rRNA gene sequencing, 16S rRNA gene sequencing plus TOF-MS (two cases), agar technique (two cases, one with growth inhibition test), polymerase chain reaction plus nucleic sequencing (two cases), antibody test (1 case), immunofluorescence test plus immunoblot assay (one case), IST2 kit (one case), and no description (one case) [2-19] (Table 2). The incubation time for the growth of *M. hominis* colonies ranged from two to five days according to previous reports [3-7, 14-16]. In this case, the colony appeared two days after inoculating the culture on blood agar. Considering that several laboratories usually culture for only 48 h, observing the medium for at least five days is recommended if *M. hominis* is suspected or if no organism is observed with Gram staining. Acridine orange staining can be helpful as an additional test if *M. hominis* is not visible on Gram staining. Owing to the challenges in using specific media and equipment for identification, *M. hominis* infections, including intracranial abscesses, are likely underreported. Thus, it is necessary to establish a well-organized collaborative system that ensures access of medical institutions to specialized facilities capable of conducting the identification and susceptibility testing of *M. hominis*.

Third, fluoroquinolones, clindamycin, and tetracyclines have been reported to exhibit enhanced in vitro antimicrobial activities against *M. hominis*. In the previous reports, the patients were successfully treated with tetracycline, lincosamide, and quinolone either as single agents or in combination [3, 4, 6-19] (Table 2). The tetracycline resistance rate varies by region and ranges from 10 to 40 % [23]. Resistance to 14- and 15-membered macrolide in *M. hominis* has been attributed to mutations in the 23S rRNA gene [24]. However, macrolide resistance tests are typically not conducted in standard laboratories. In this case, colony growth was insufficient to determine the MIC values. As the tetracycline resistance status was unknown, we avoided using tetracycline and chose a combination therapy with levofloxacin and clindamycin. Because infections caused by *M. hominis* have limited treatment options, it is recommended to conduct antibiotic susceptibility testing, although the test could not be conducted in this case because of poor culture growth.

## Conclusions

We encountered a case of *M. hominis* intracranial abscess following a traffic injury, diagnosed by characteristic colony morphology obtained through extended culture. The patient was successfully treated with a combination therapy of levofloxacin and clindamycin. When bacteria are suspected to be the cause of an intracranial abscess with a risk of *M. hominis* infection, and Gram staining is inconclusive, considering *M. hominis* as one of the potential causative pathogens and conducting extended culture is important.
